# Network analysis of interactions of rumination and anxiety on smartphone dependence symptoms

**DOI:** 10.3389/fpsyt.2025.1506721

**Published:** 2025-02-04

**Authors:** Sen sen Zhang, Shao hong Yong, Jia tai Chen

**Affiliations:** ^1^ Faculty of Business Administration, Guangzhou Institute Of Science And Technology, Guangzhou, China; ^2^ Department of Psychology, Institute of Teacher Education, Ningxia University, Yinchuan, China; ^3^ Business School, University of Exeter, Exeter, United Kingdom

**Keywords:** network analysis, rumination, anxiety, smartphone dependence, preservice teachers

## Abstract

**Objective:**

Rumination and anxiety have been posited as correlates of smartphone dependence (SPD). However, little is known regarding how the components of both affect SPD symptoms at subtle levels. Therefore, we used the network analysis approach to identify the connections at a micro level to provide possible interventions for reducing SPD symptoms.

**Methods:**

Using symptom-level network analysis, we used the ruminative response scale-10, the generalized anxiety disorder scale-7, and the mobile phone addiction index scale-17 to investigate Chinese preservice teachers (*M*_age_ = 21.1, *N* = 1160). Subsequently, we estimated a graphical lasso correlation network for these teachers, which encompassed rumination components, anxiety components, and SPD symptoms. Specifically, the central and bridge centralities within the network structure were examined for the impacts of rumination and anxiety on SPD symptoms.

**Results:**

The three intracluster connections of rumination, anxiety, and SPD were tighter than the intercluster, with structural connections in rumination and anxiety networks closer than the triggered SPD symptoms cluster. Importantly, reflection reactions towards “write down what you are thinking and analyze it” (a component of rumination) were identified as a central and bridging node that might be a target for intervention for SPD symptoms.

**Conclusion:**

We identify potential edge-bridging rumination and anxiety on SPD and locate highly central components within each cluster via network analysis.

## 1 Introduction

Smartphone overuse could not be ignored (1), which was typified as a behavior of non-chemical dependence (2) and a major predictor of smartphone dependence (SPD; 3). 15% of the youth in the US were classified as severely SPD, and 46% of smartphone users said they “could not live without.” The global SPD rate was 28.3% (4), with notably higher prevalence levels among college students than other adults. In particular, during COVID-19, countries started to implement mitigation strategies such as staying home, shutting down workplaces, and limiting mobility (5), which reached a 41.93% prevalence rate in the group of Asian students (6). Simultaneously, the quality of rural education is challenged by a global teacher shortage (7), and the educational system in both urban and rural China is very imbalanced (8, 9). Additionally, the urban-rural educational gap has been worsened by disparities in higher education affordability (10, 11). Thus, the Chinese government implemented the Free Teacher Education program to encourage young graduates to pursue careers as teachers and to enhance education in disadvantaged rural areas through free college education and guaranteed jobs (12). It is similar to outstanding programs like Teach for America, Teach First in the United Kingdom, and Teach for Australia, which attempt to fill vacant teaching posts with skilled instructors to alleviate educational inequity (13). However, due to the policy assurance that participants could become teachers if they were successfully recruited, their SPD during the educational stage might be exacerbated to some extent (11).

### 1.1 Theoretical perspectives on smartphone dependence

Dependence is defined as adapting to the availability of something, while addiction refers to the compulsive use of something regardless of harmful consequences (3). Choliz (14) defined four common factors of dependence and addiction as lack of control, abstinence, tolerance, abuse, and disruption with other activities. Meanwhile, Sansone and Sansone (15) noted that misuse, abuse, dependence, and addiction were still not distinguished. Some studies used “addiction” and “dependence” interchangeably in the same study without clearly distinguishing them (e.g., 16). Therefore, we use SPD in the present study with caution to describe that the reinforcement of smartphone use might lead to problematic symptoms. Given the high prevalence and negative daily life consequences of SPD, which were increasingly noticed by mental health professionals, and would be necessary to analyze the mechanisms underlying SPD. The compensatory internet use theory (CIUT; 17) and the interaction of person-influence-cognition-execution (I-PACE; 18) theoretical models provided a robust theoretical foundation for understanding the causes of SPD. People, according to the CIUT, engage in excessive technology overuse to mitigate negative emotions. For instance, individuals might increase SPD in response to distress and negative emotions associated with unexpected events in daily life, such as enforced seclusion. Meanwhile, the empirical discovery-based I-PACE model revealed individual dispositions (e.g., cognitive responses and emotions) likely contributed to vulnerabilities in the occurrence and maintenance of SPD (17, 18).

### 1.2 Rumination, anxiety, and smartphone dependence

Rumination and anxiety may be risk factors for SPD (19–21). For example, policy-enforced mitigation strategies may increase social isolation and loneliness (22, 23), which would trigger rumination and anxiety, leading to the likelihood of psychiatric disorders (24, 25). That was because, on the one hand, rumination was a cognitive process involving repetitive reflections on negative experiences and feelings, related to psychopathology (26). It comprised repetition and passive attention on distressing symptoms and potential sources or outcomes from them (21, 27) with the inability to eliminate negative information from memory, which was considered as a deficit in control processes (28, 29). Therefore, growing research has focused on the relationship between rumination and cognitive control (e.g., SPD in daily life; 20, 30, 31). On the other side, rising uncertainty caused by COVID-19 could cause anxiety, and rumination was also a significant trigger of anxiety symptoms (25, 32), which would expose individuals to negative emotions fueled by a perceived threat, injustice, and self-loss (33, 34). Such individuals were susceptible to slipping into repetitive and negative thoughts about distress, strangling communication, and curbing dynamic behaviors (20, 21) in response to unfavorable events. It facilitated deeper processing of affective-related patterns and amplified emotional states (27, 32), aggravating symptoms of anxiety by worsening a “sense of uncontrollability focused on the possibility of future threat, danger, or other potentially negative events” (35). Smartphone users with higher degrees of anxiety showed more SPD (19). Finally, according to the I-PACE, habitual behavior development was the result of interactions among susceptibility variables (e.g., social isolation), cognitive responses to specific stimuli (e.g., rumination) and feelings (e.g., anxiety), and executive functions (e.g., decision or inhibitory control), in which associations contribute to the chronic behavior development (18). Meanwhile, the CIUT focused on the overuse of digital technology as a compensatory coping strategy. Existing studies reported that the CIUT and I-PACE models could be used to explain SPD (33, 36, 37), and recent research has explored the psychopathological constructs that underpin problematic smartphone use through the I-PACE (38).

### 1.3 Network analysis

Despite the substantial contributions provided by previous studies, a recent investigation revealed that an individual’s intolerance of uncertainty significantly predicted SPD, in this context, rumination and anxiety were identified as mediators (11). A nuanced approach to understanding the differences between rumination and anxiety to specific SPD symptoms is lacking. SPD might involve heterogeneous states of different symptoms (e.g., “inability to control craving,” “feeling anxious and lost,” “withdrawal/escape,” and “productivity loss”), with each differing relative weight (1, 39). Similarly, rumination and anxiety were multidimensional constructs (27, 40). These different ingredients might have various actions on the symptoms (11). Therefore, ignoring structural or symptom heterogeneity would be problematic, as it might prevent a more microscopic understanding of symptom interactions from a “small world” perspective (41, 42). Specifically, the appearance of one symptom is considered to increase the probability of the emergence of interrelated symptoms, in turn, which could lead to episodes of illness. It differed from the latent variable model, which supposed unobservable latent variables resulting in observable symptoms (41). For example, individuals with SPD show different association strengths with rumination (30) and anxiety (11, 32, 33), indirectly confirming this drawback. Research in psychopathology would probably benefit from “moving beyond disorder-level analysis to a more fine-grained symptom-level analysis” (38, 43). Networks analysis, a symptom-oriented graphical approach, conceptualizes psychopathology as complex systems with nodes that interact and strengthen with each other through underlying causal connections (i.e., edges). It has provided the ability to visualize complicated relationships across psychological structures at a subtler level (e.g., components and symptoms), which could contribute to researchers elucidating the mechanisms that underlie the symptoms’ associated components and developing more accurate and targeted interventions (41, 44).

### 1.4 The present study

To our knowledge, no studies have examined how individual components of rumination and anxiety lead to specific SPD symptoms. Furthermore, even fewer analyses have been conducted in the particular group of preservice teachers. Educational inequities were prevalent worldwide before the pandemic, and the Chinese school system was also highly unbalanced (8, 13, 45). Focusing on them in economically underdeveloped areas and SPD symptoms would be significant for educational development. Therefore, the present study used network analysis to develop a model of the component-symptom associations across rumination and anxiety on preservice teachers’ SPD symptoms. Specifically, we studied (1) the unique associations among rumination and anxiety components with SPD symptoms, (2) the influential nodes that maintain the network structure, and (3) the most influential nodes that bridge the rumination and anxiety clusters to SPD symptom clusters.

## 2 Methods

### 2.1 Participants

The participants in this study were a group of preservice teachers who were forced to isolate on college campuses due to epidemic control measures. They were drawn from six universities in West China, and 1,610 valid matching responses were retained for analysis.

In **Table 1**, we present the demographic characteristics of the study’s participants, their average age was 21.11 years (*s* = 2.13); 827 (51.4%) were female, and 783 (48.6%) were male. Additionally, the participants’ birthplace consisted of urban (30.0%) and rural (69.9%) areas, while their academic backgrounds varied, with 45.1% majoring in Science and Engineering, 40.4% in Humanities and Social Sciences, and 14.5% in Arts and Sports. Furthermore, we included information regarding family and relationship factors, such as whether they were the only child (13.9%), and the educational background of their fathers and mothers. Lastly, their romantic relationship lengths were recorded, with 56.4% reporting no current romantic relationship, and the remaining participants were distributed across different relationship durations. For detailed demographic information, see **Table 1**.

**Table 1:** Demographic characteristics of participants.

Demographic variables		*N* (%)	Demographic variables		*N* (%)
Gender	Male	783(48.6)	Grade	One	463(28.8)
	Female	827(51.4)		Two	668(41.5)
Birthplace	Urban	483(30.0)		Three	238(14.8)
	Rural	1126(69.9)		Four	241(15.0)
OOC		223(13.9)	FEB	High School and Below	1428(88.7)
SC	Science and Engineering	726(45.1)		College	92(5.7)
	Humanities and Social Sciences	651(40.4)		Bachelor	78(4.8)
	Arts and Sports	233(14.5)		Graduate and above	12(0.7)
RRL	None	908(56.4)	MEB	High School and Below	1476(91.7)
	Less than one year	340(21.1)		College	76(4.7)
	1-2 years	196(12.2)		Bachelor	47(2.9)
	2-4 years	104(6.5)		Graduate and above	11(0.7)
	More than four years	62(3.9)			

*N* = 1610. SC, Subject classification; OOC, Only one child; FEB, Father’s Educational Background; MEB, Mother’s Educational Background; RRL, Romantic Relationship Length.

### 2.2 Measures

We surveyed Chinese preservice teachers using a demographic information form, ruminative response scale, generalized anxiety disorder scale, and mobile phone addiction index scale.

#### 2.2.1 Demographic information form section

To improve appropriateness and transparency in using control variables, our demographic questionnaire followed recommendations by Bernerth and Aguinis (46), including specific control variables based on previous research. Specifically, gender, grade, subject classification, romantic relationship lengths, and birthplace were included, along with information regarding family and relationship factors, such as whether they were the only child and the educational background of their parents. By incorporating these control variables into our data collection process, we aimed to account for potential sources of variance and enhance the validity of our results.

#### 2.2.2 Ruminative response scale-10

The RRS-10 scale was designed by Trapnell and Campbell (34) to assess ruminant thoughts on negative self-focus triggered by feelings of self-threat. It consisted of “reflection” and “brooding” two subscales, and scores were calculated by summing all items on a 4-point scale (1 = not at all to 4 = almost always), with higher scores indicating higher levels of rumination. Furthermore, RRS translated into Chinese for research purposes were used, showing good validity and internal reliability (29). In our study, the Cronbach’s alphas were 0.852, 0.825, and 0.911 for the subscale and total scale, respectively, demonstrating good internal consistency (McDonald’s omega = 0.926), and the fitting index (RMSEA = 0.075, CFI = 0.947, TLI = 0.930, SRMR = 0.036) was ideal.

#### 2.2.3 Generalized anxiety disorder scale-7

Anxiety was assessed via the one-dimensional GAD-7 (40) that used four options of seven items to describe the frequency of each symptom experienced over the past two weeks (0 = not at all to 3 = nearly every day). The items were summed to obtain a total score, with higher scores indicating more severe symptoms. It exhibited good consistency and reliability in the Chinese context (31). In our study, the Cronbach’s alphas was 0.936, McDonald’s omega was 0.948, and the fitting index (RMSEA = 0.058, CFI = 0.980, TLI = 0.970, SRMR = 0.022) was ideal.

#### 2.2.4 Mobile phone addiction index scale-17

The MPAI-17 scale was developed by Leung (39) based on previous research (47). It was used to identify phone addiction symptoms and served as a comprehensive assessment, meeting the DSM of Mental Disorders assumptions about substance addiction symptoms. It consisted of four subscales, namely “inability to control craving, feeling anxious and lost, withdrawal/escape, and productivity loss.” It was a 5-point scale (1 = not at all, 2 = rarely, 3 = occasionally, 4 = often, and 5 = always). Higher mean scores on each subscale indicated more severe addiction, which has been widely used in measuring SPD (48, 49). In the present study, the Cronbach’s alphas were 0.867, 0.862, 0.862, 0.840, and 0.935 for the subscale and total scale, respectively, demonstrating good internal consistency (McDonald’s omega = 0.943). Furthermore, the fitting index proved to be ideal by CFA (RMSEA = 0.065, CFI = 0.922, TLI = 0.906, SRMR = 0.048).

### 2.3 Procedure

Data were collected in two ways. One was in the classroom utilizing paper questionnaires in June 2022, and the other was collected online through *Wenjuanxing*, a Chinese online survey platform from July to September, and both ways used the same questionnaire. This multi-sample design delivered credible and reproducible empirical evidence that extended the external validity of experimental findings and responded to the replication crisis in psychological science research (50, 51). During the data collection procedure, participants were told to participate voluntarily, and informed consent was obtained from all. Participants were asked whether there was a period when they were obliged not to leave their residence due to epidemic policies. If the answer were “no,” they would not be invited to complete the following questionnaire. Additionally, the data collection was mainly based on a self-reported method, thus during actual implementation, after consultation with the participants and counselors, both offline and online data collection were measured in the classroom before weekly class meetings for an objective and accurate data collection process to control the impact of the external context impact if possible. We distributed 600 paper questionnaires and returned 560, with a response rate of 93.3%; 1400 electronic questionnaires were distributed and received a response rate of 84.1% for 1,082 returns. After screening out incomplete and invalid questionnaires, 1610 samples were available for the following analysis.

### 2.4 Data analysis

We used SPSS 25.0 for descriptive statistics, correlation analysis, etc., and network analysis ran on R version 4.1.0. First, because items contained 4-point ordinal scales, the graphical least absolute shrinkage, and selection operator in the *qgraph* package build the network structure (42, 52). Due to the massive estimated parameters in the network (i.e., 34 nodes need to estimate 595 parameters: 34 threshold parameters and 34*33/2 = 561 pairwise correlation parameters), it might lead to some false positive edges. Therefore, all edges in the network were reduced by the graphical lasso (glasso; 52, 53) algorithm, and the minor edges were set precisely to zero, which maximized the fit and explained the covariances across nodes with the fewest possible edges. Further, the hyperparameter *γ*, controlling the weighing between false positive edges and removed true ones, was set to 0.5 (54), along with the extended Bayesian Information Criterion model selected to obtain a parsimoniously accurate structure. In the network, the nodes represent items, and the edges refer to the correlations, with green for the positive direction and red for the negative; its width indicates the strength of the correlations.

Second, node centrality indices (i.e., strength, betweenness, and closeness) were weighed. Node strength is calculated by summing all the weights of edges connected directly to it, with a higher-strength node sharing a stronger direct connection with many other nodes. As increasing evidence suggested that betweenness and closeness would not be reliable (53), we calculated the node’s expected influence besides the strength, which simultaneously considers the positive and negative edge, and gives sums of edge weights of nodes, showing a better way to identify influential nodes with negative edges (55). Furthermore, to identify critical nodes for cluster connectivity, we used the *networktools* package to computer the bridge expected influence based on edge weights from a given node to other clusters (56). Those nodes having a higher bridge expected impact are assumed to perform a more significant role in activating nodes from the opposite clusters. A rigorous method was used to determine the centrality and bridge nodes with a blind 85th percentile cutoff on the values of both node and bridge expected influence to avoid possible confirmation bias in the interpretation of centrality statistics.

Finally, we used the *bootnet* package bootstrapping method to evaluate the estimation accuracy and robustness of the network (53). On the one side, the accuracy estimation was performed by drawing bootstrap confidence intervals (*CIs*) from each edge weight. Higher *CIs* of overlapping edge weights indicate lower accuracy of the graphical depiction. On the other side, centrality index stability was estimated by bootstrap resampling of a subset of the total samples. Specifically, it shows how the centrality index changes as the proportion of the sample subset decreases (e.g., comparing the whole samples with only 30% of them). The more rapidly the centrality shifts with decreasing sample proportions, the less stable it is. The correlation stability coefficient shows the maximum acceptable degree of sample reduction, with good above 0.70, acceptable above 0.50, and minimum not less than 0.25 (53).

## 3 Results

### 3.1 Descriptive statistics

We examined the means and variability of all items (see **Supplementary Table A**) and reported the correlation coefficients between variables’ dimensions (see **Table 2**), according to the recommendations of Fried (57), a higher score indicated a stronger propensity for the trait. **Table 2** displays the correlation coefficients between the variables dimensions. Anxiety is moderately positively correlated with *brooding* (r = .333, *p* < 0.01) and strongly positively correlated with *reflection* (r = .815, *p* < 0.01), indicating that higher anxiety levels are associated with increased *brooding* and heightened *reflection* tendencies. Furthermore, anxiety is positively correlated with *inability to control craving* (r = .475, *p* < 0.01), *feeling anxious and lost* (r = .490, *p* < 0.01), *withdrawal/escape* (r = .360, *p* < 0.01), and *productivity loss* (r = .406, *p* < 0.01), revealing that elevated anxiety levels are associated with greater *inability to control cravings*, intensified *feeling anxious and lost*, a heightened inclination toward *withdrawal/escape*, and strengthen *productivity loss*. Moreover, brooding and reflection are both positively correlated with inability to *control craving* (*brooding*: r = .362, *p* < 0.01; *reflection*: r = .440, *p* < 0.01), *feeling anxious and lost* (*brooding*: r = .327, *p* < 0.01; *reflection*: r = .397, *p* < 0.01), *withdrawal/escape* (*brooding*: r = .343, *p* < 0.01; *reflection*: r = .386, *p* < 0.01), and *productivity loss* (*brooding*: r = .340, *p* < 0.01; *reflection*: r = .417, *p* < 0.01). These findings provide a concise overview of the associations between these variables, offering support for further focus on micro-level relationships in subsequent analyses.

**Table 2:** Mean, standard deviation (SD), and correlation between each variable selected in the present study.

Variable	*M*	*SD*	1	2	3	4	5	6	7
Anxiety	1.49	0.66	1						
Brooding	2.21	0.73	.333^**^	1					
Reflection	1.98	0.69	.439^**^	.815^**^	1				
Inability to control craving	1.92	0.80	.475^**^	.362^**^	.440^**^	1			
Feeling anxious and lost	1.98	0.92	.490^**^	.327^**^	.397^**^	.693^**^	1		
Withdrawal/escape	2.21	1.07	.360^**^	.343^**^	.386^**^	.579^**^	.672^**^	1	
Productivity loss	2.07	1.06	.406^**^	.340^**^	.417^**^	.682^**^	.658^**^	.618^**^	1

*N* = 1610; ^**^ *p* < 0.01; all tests were two-tailed.

### 3.2 Network structure

**Figure 1:**
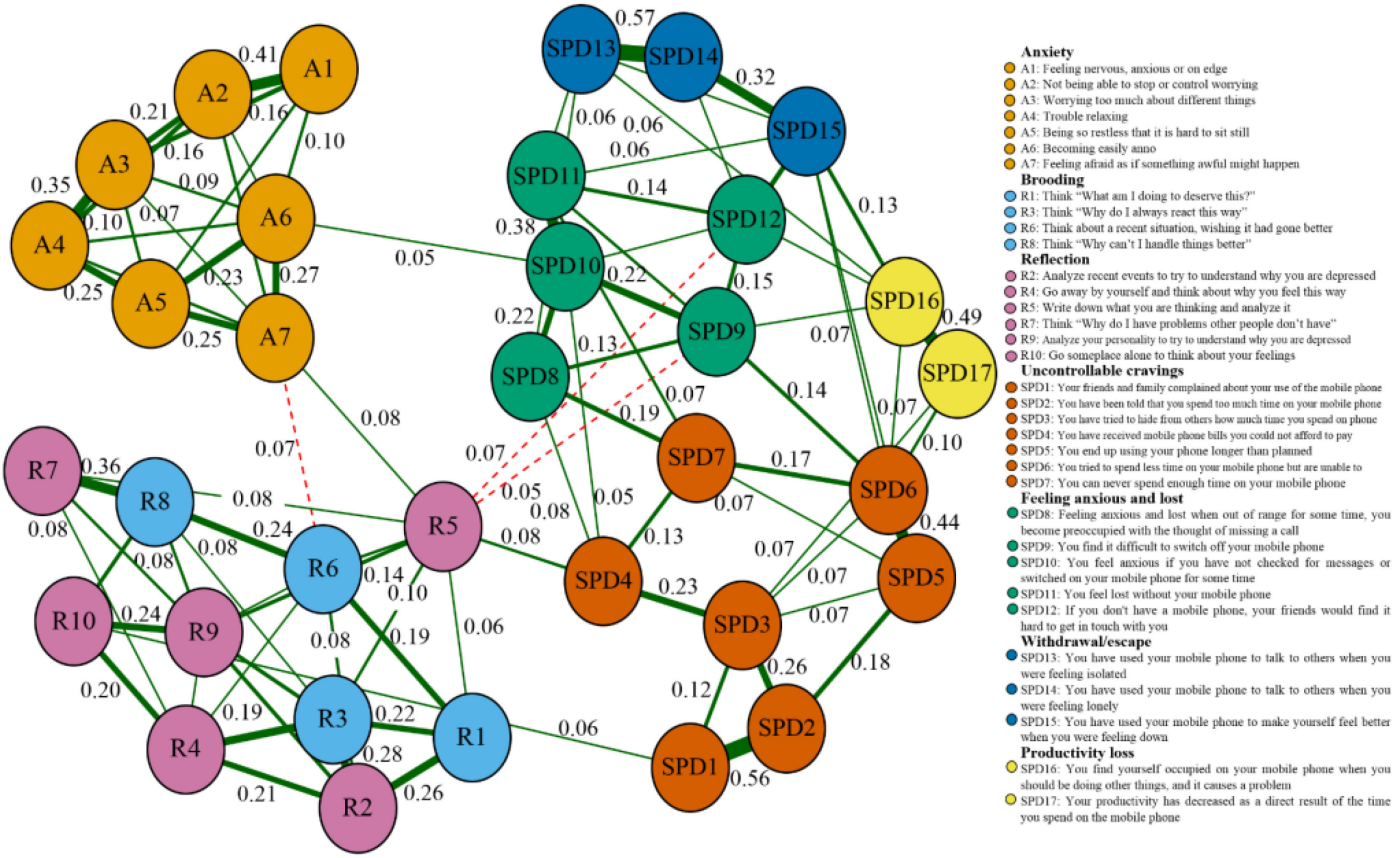
Network structure for symptoms of anxiety, rumination, and SPD (cut value = 0.03; *N* = 1,610). Red dashed edges represent negative correlations, and solid green edges represent positive ones; the thickness maps to the magnitude of associations.

The network structure shows that items of anxiety and rumination were mainly clustered with the compact connectivity, compared to items assessing SPD were more distant and less connected (see **Figure 1**). Within the network, 34 items were displayed, 252 (44.9%) of 561 possible edges (weights range from -.073 to.569), and more positive edges (n = 221) than negative ones (n = 31) were observed in general. Furthermore, among cluster edges, the strongest were R5 (“Write down what you are thinking and analyze it”) and SPD3 (“You have tried to hide from others how much time you spend on your mobile phone”; edge weight.092); R5 (“Write down what you are thinking and analyze it”) and SPD4 (“You have received mobile phone bills you could not afford to pay”; edge weight = .078). The strongest negative associations between cluster edges were A7 (“Feeling afraid as if something awful might happen”) and R6 (“Think about a recent situation, wishing it had gone better”; edge weight = -.073); followed by R5 (“Write down what you are thinking and analyze it”) and SPD12 (“If you don’t have a mobile phone, your friends would find it hard to get in touch with you”; edge weight = -.069). Meanwhile, the cluster of SPD symptoms was internally intensive, and all associations were majority positive (weights range from -.020 to.569).

### 3.3 Centrality indices

**Figure 2:**
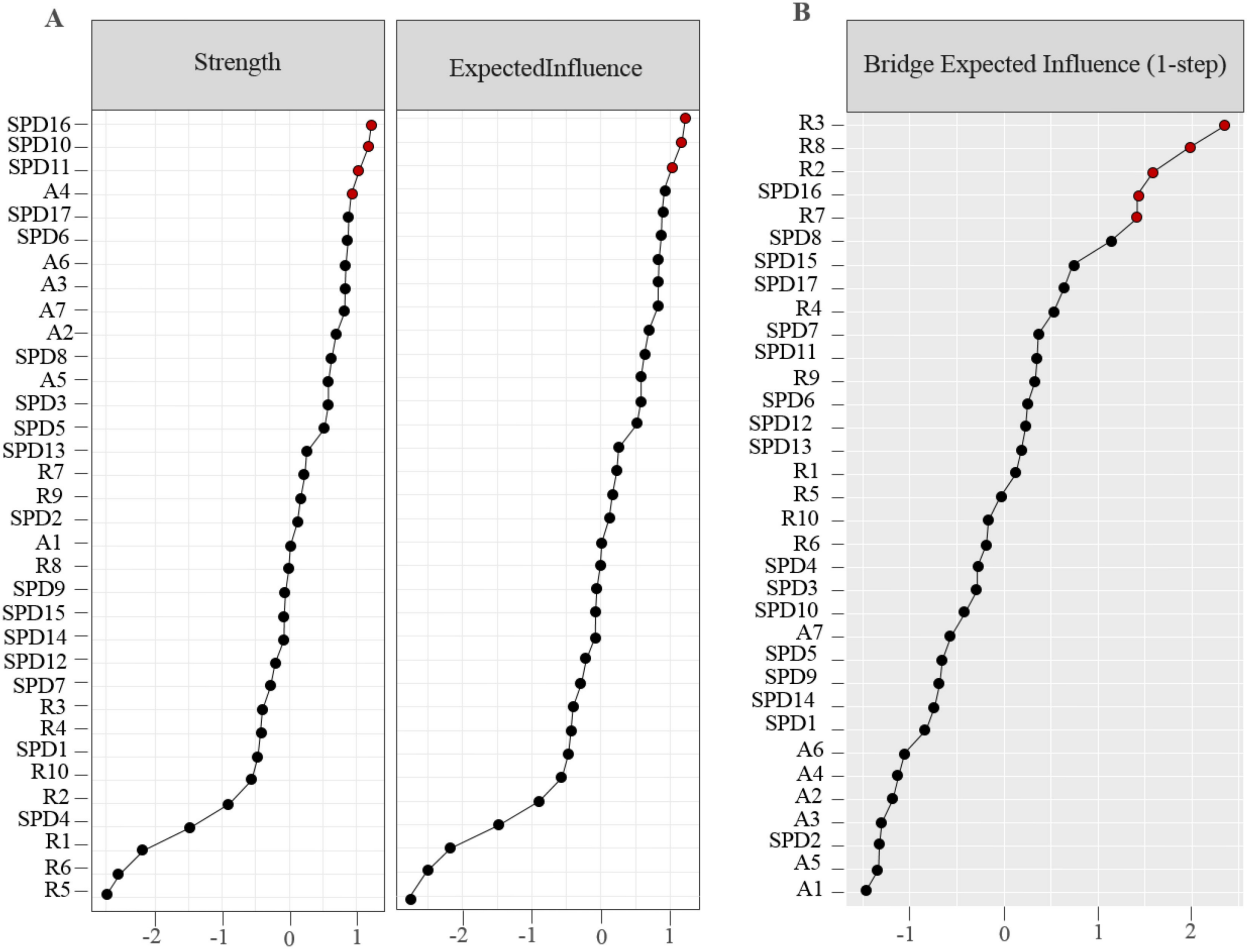
Centrality indices describe **(A)** strength and expected influence; **(B)** bridge expected influence, and are displayed as standardized value z-score.

The sample strength and expected influence values are shown in **Figure 2A**. Some items showed a higher strength, e.g., items A4 (“Trouble relaxing”; strength value = 1.171), R3 (“Think why do I always react this way”; strength value = 1.226), R8 (“Think why can’t I handle things better”; strength value = 1.175), SPD2 (“You have been told that you spend too much time on your mobile phone”), and SPD6 (“You have attempted to spend less time on your mobile phone but are unable to”; strength value = 1.189), which indicated these items were the more cored nodes. Meanwhile, items R2 (“Analyze recent events to try to understand why you are depressed”; expected influence value = 1.027), R3 (“Think why do I always react this way”; expected influence value = 1.137), R7 (“Think why do I have problems other people don’t have”; expected influence value = 0.905), and R8 (“Think why can’t I handle things better”; expected influence value = 1.084) showing a great expected influence, were recognized as the central nodes of the symptom network. Finally, five bridging nodes are R2 (“Analyze recent events to try to understand why you are depressed”; bridge expected influence =.663), R3 (“Think why do I always react this way”; bridge expected influence =.824), R7 (“Think why do I have problems other people don’t have”; bridge expected influence =.627), R8 (“Think why can’t I handle things better”; bridge expected influence =.740), and SPD16 (“You find yourself occupied on your mobile phone when you should be doing other things, and it causes a problem”; bridge expected influence =.627) that had the greatest bridge expected influence (Details see **Figure 2B**).

### 3.4 Accuracy of the networks

**Figure 3:**
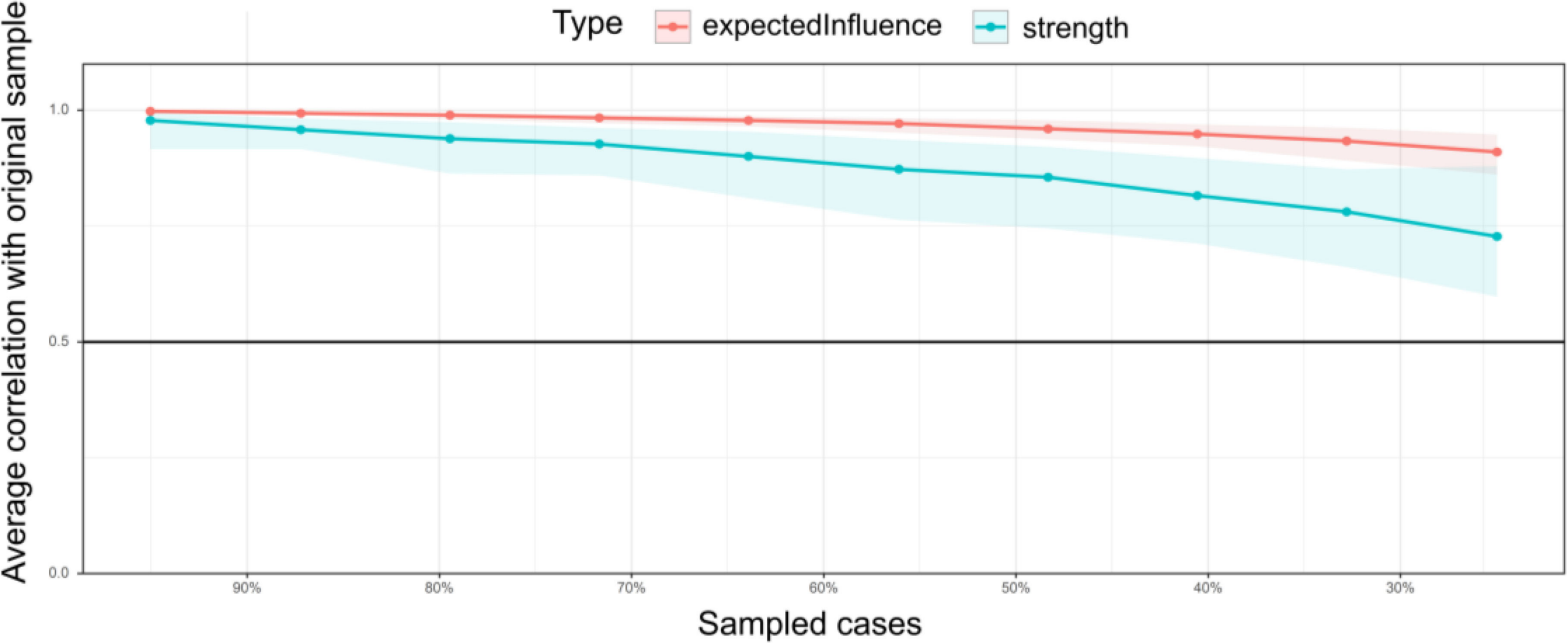
Node strength and stability of expected influence. The solid bars represent the average correlation of node strength and expected influence in the total and subsamples, with the shaded area describing the 2.5th to 97.5th quartile.

The relatively narrow 95% *CIs* of the bootstrap indicate that the edges of the network structure would be judged accurately (see **Supplementary Figure S1**), and we performed bootstrap difference tests for edge weights, node strength, expected influence, and bridge expected influence separately (see **Supplementary Figures S2**-**S5**). These methods were based on the recommendations of Epskamp et al (53). In addition, the correlation stability coefficients for both node strength and expected influence decreased smoothly in line with the reducing proportion of sampled cases, and both (strength value = .594 and expected influence value = .750) were greater than 0.50, with *CIs* above 0.25 (see **Figure 3** for details; 53). It demonstrated that the centrality indices of both node strength and expected influence were sufficiently stable.

## 4 Discussion

Existing research suggests that rumination and anxiety may exacerbate SPD symptoms. In our study, we used network analysis to delve deeper into the intricate relationships between rumination and anxiety to specific SPD symptoms in a preservice teacher population. Our initial findings revealed that the three intracluster connections were tighter than intercluster ones, similar to previous findings (38, 58), together with structural connections in rumination and anxiety networks closer than clusters triggering SPD symptoms. On the one side, it showed a greater association between rumination and anxiety, expanding more microscopically based on previous studies (21, 32). On the other side, the two factors were also indicated as causal drivers of SPD symptoms, which followed the theoretical hypothesis of the CIUT that perceived regurgitation of specific stimuli and adverse emotional anxiety reactions preferred reducing their inhibitory control and engaging in short-term behaviors (e.g., smartphone use). It might be a maladaptive response strategy acquired after being disturbed by external situations (e.g., social isolation; 38), which aligned with previous findings that higher SPD correlated with greater rumination (20) and anxiety (31).

In a closer examination of the rumination and anxiety clusters, the most potent negative edge was A7 (“Feeling afraid as if something awful might happen”) and R6 (“Think about a recent situation, wishing it had gone better”), and the positive one was A3 (“Worrying too much about different things”) and R10 (“Go someplace alone to think about your feelings”), which conformed to previous studies (26, 32). Nodes R2 (“Analyze recent events to try to understand why you are depressed”), R3 (“Think ‘Why do I always react this way?’”), R7 (“Think ‘Why do I have problems other people don’t have?’”), and R8 (“Think ‘Why can’t I handle things better?’”), were not only important expected influence nodes but also critical bridge nodes in the susceptibility factor network structure. Additionally, these nodes were separately clustered as reflection and brooding. The former components showed purposefully turning inward for cognitive problem-solving to relieve the status quo; in contrast, the latter was a passive comparison of the status quo and unmet standards (27). Meanwhile, for the SPD cluster, we found that SPD1 (“Your friends and family complained about your use of the mobile phone”) was closely related to SPD2 (“You have been told that you spend too much time on your mobile phone”), and SPD13 (“You have used your mobile phone to talk to others when you were feeling isolated”) to SPD14 (“You have used your mobile phone to talk to others when you were feeling lonely”), respectively. Both of those strong association was related to the fact that they belonged to SPD symptoms (39); despite the co-occurrence of feeling anxious with escape and uncontrollable craving, we need to consider the potential confounding influence that such correlations might derive from different causal sources. Overall, we presented symptoms based on micro-specific network structures as critical features, which could provide feasible intervention and treatment strategies, given that these features probably would have a more significant impact or lead to other ones (41, 44, 59).

Examining the centrality and node bridges within our network analysis reveals the specific functions performed by the different components of rumination and anxiety in SPD development and maintenance. We identified that the most robust node linking susceptibility factors and SPD symptom clusters was R5 (“Write down what you are thinking and analyze it”; bridge strength value = .836). It tightly connected positively with A7 (“Feeling afraid as if something awful might happen”) within the susceptible network component, positively associated with SPD3 (“You have tried to hide from others how much time you spend on your mobile phone”) and SPD4 (“You have received mobile phone bills you could not afford to pay”), and negatively correlated with SPD9 (“You find it difficult to switch off your mobile phone”) and SPD12 (“If you don’t have a mobile phone, your friends would find it hard to get in touch with you”), respectively. Moreover, R5 was the reflective component, SPD3 and SPD4 were uncontrollable cravings components, and SPD9 and SPD12 were feeling anxious and lost components. It was consistent with the assumptions of the I-PACE, because some vulnerability variables cause individuals to experience higher adverse emotional reactions. Such individuals struggle to relax and might make phone access a maladaptive coping strategy, contributing to SPD as a habitual behavior. Our results extended previous research (38, 60) that feeling anxious and lost contributed to SPD core symptoms. Furthermore, R5 and its associated SPD symptom components might be targets for an examination and intervention in various mental health conditions that meet current diagnostic model recommendations in psychopathology (44, 61). R5, which has alluded to the main principle of diary therapy may reduce anxiety, promote psychological recovery, and improve the quality of life by recording and analyzing unfavorable events, although the subjectivity of the recorder is different (62); an earlier study found that high ruminants using a self-compassionate writing task reduced melancholy more than low ruminants adopting a distraction activity in response to unpleasant emotions induced by unfavorable experiences (63). Still, we tested a larger group of participants at different time points, and the results would be more realistic and reliable; the centrality of nodes between cross-sectional and participant networks might not be fine-define (64), which requires further research.

Although our study revealed novel findings, some limitations need to be noted. First, our sample is specific to pre-service teachers in China, a subset of community samples, which may inherently exhibit lower levels of psychopathology (57), and future research could benefit from examining selected groups with varying diagnostic profiles. Second, timescales are important for thinking about how different levels, such as physiological and psychological, interact with each other (41). Although we have collected data at different time points for preservice teachers, it would be helpful to lengthen time intervals further and measure each individual repeatedly to capture the fluctuations in understanding psychopathology (65). Additionally, our cross-sectional design, coupled with the challenges posed by data collection during the COVID-19 pandemic, may hinder causal inference. Future longitudinal studies could be recommended to validate our findings and elucidate causal relationships between variables (66). Finally, the uncertainty caused by sampling variation needs to be considered. We intentionally increased participants and collected data in different regions. Still, sampling variations and power limitations should not be ignored (64), and future research should also explore the potential influence of demographic factors such as gender, birthplace, and socioeconomic status on the network structure of psychopathology, as these factors may play a role that was not significantly evident in the present study, particularly in the context of the pandemic.

The present findings have significant implications for interventions aimed at disrupting the development and co-occurrence of disorders by identifying central and bridging nodes. Cuthbert and Insel (61) and Fried et al. (59) have highlighted the potential of early intervention strategies informed by such identifications. Based on our findings, R5 emerged as a central and bridging node that might be a potential target for early intervention in SPD. Previous studies have demonstrated favorable outcomes in related symptoms following the reduction of anxiety (19). Encouraging pre-service teachers to adopt diary therapy and self-compassionate writing as coping mechanisms for negative feelings spurred by unfavorable events could be a strategic approach for educators (62, 63). These techniques encourage reflective thinking, a process generally seen as beneficial but one that may require careful management in the context of SPD. Reflective thinking, particularly when focused on negative experiences, can lead to repetitive reflections and deep processing of emotion-related patterns (27, 32). This, in turn, may exacerbate anxiety (35).

To mitigate the adverse effects of reflective thinking on SPD symptoms, we propose incorporating brief mindfulness training programs. Such programs have been shown to reduce negative feelings and, consequently, SPD symptoms (26). Mindfulness training encourages individuals to observe their thoughts and feelings without judgment, thereby reducing the tendency to ruminate on negative experiences. This approach not only addresses the symptoms of SPD but also has the potential to contribute to the reduction of substance abuse, as suggested by previous studies (60, 67). Furthermore, given the importance of SPD16 (“You find yourself occupied on your mobile phone when you should be doing other things, and it causes a problem”; bridge strength value = .627) as a significant bridge node within the SPD symptom clusters, our findings indicate that interventions targeting this node may be particularly effective in disrupting the spread of SPD symptoms. However, SPD16 was not directly influential in engaging rumination and anxiety with SPD, differing slightly from Liu et al. (38) findings, which revealed “withdrawal/escape” as the most robust pathway connecting uncertainty intolerance and problematic smartphone use. This suggests that various factors may trigger SPD symptoms at different key nodes, necessitating early identification and node-specific interventions. In conclusion, our findings highlight the potential of node-specific interventions targeting coping skills to reduce SPD symptoms. Future research should further explore the effectiveness of these interventions, including mindfulness training and reflective thinking management, in diverse populations and with various triggering factors.

## 5 Conclusion

Through the network analysis approach, we enhance the insight into associations of preservice teachers’ rumination and anxiety components linked to SPD. Specifically, first, tighter intracluster connections within rumination and anxiety networks compared to intercluster ones, suggesting tighter links within the same factors. Second, we identify potential edge-bridging ruminants and anxiety within each cluster, which supports the theoretical hypothesis of the compensatory internet use theory (CIUT) and would provide clues for future research to develop theoretical understanding and interventions. Finally, we identify potential edge-bridging rumination and anxiety on SPD, specific nodes within the rumination and anxiety clusters were characterized as influential and critical bridge nodes in the susceptibility factor network structure. These nodes represented distinct cognitive processes, such as reflection and brooding, and were associated with core SPD symptoms, which aligned with the assumptions of the interaction of the person-influence-cognition-execution (I-PACE) model, indicating that these nodes might be a potential target for intervention and treatment in SPD. Notably, the centrality analysis revealed that node R5 (“writing down what you are thinking and analyzing it”), played a significant role in linking susceptibility factors and SPD symptom clusters, positively associated with uncontrollable cravings and negatively correlated with feelings of anxiety and loss. The practical solution of self-compassionate writing aligns with the principles of diary therapy, which has been shown to reduce anxiety, promote psychological recovery, and improve the quality of life by recording and analyzing unfavorable events. In summary, our study has shed light on the intricate relationships between rumination, anxiety, and SPD in preservice teachers. The identification of central nodes like R5 provides valuable insights into theoretical and practical implications for understanding and relieving SPD.

## Data Availability

The raw data supporting the conclusions of this article will be made available by the authors, without undue reservation.
