# Supplementary material for: Network analysis of interactions of rumination and anxiety on smartphone dependence symptoms
Source: Front Psychiatry. 2025 Feb 4;16:1506721. doi: 10.3389/fpsyt.2025.1506721 (PMC11832651; doi:10.3389/fpsyt.2025.1506721)
Supplement: Supplementary file 1 [file DataSheet1.pdf]

Supplementary Materials

Table A. Summary of mean, standard deviation, skewness, and kurtosis of each variable selected in the present networks.

Results of the accuracy assessment of the network structure.

1. Figure S1. Accuracy of edge weights.
2. Figure S2. Bootstrapped difference test for edge weights.
3. Figure S3. Bootstrapped difference test for node strength.
4. Figure S4. Bootstrapped difference test for node expected influences.
5. Figure S5 Bootstrapped difference test for node bridge expected influences.

Table A | Summary of mean, standard deviation, skewness, and kurtosis of each variable selected in the present networks.

Node	Items	*M*	*SD*	Skewness	Kurtosis
R1	Think “What am I doing to deserve this?”	2.20	0.86	0.46	-0.34
R2	Analyze recent events to try to understand why you are depressed.	2.11	0.90	0.47	-0.54
R3	Think “Why do I always react this way?”	2.10	0.88	0.45	-0.51
R4	Go away by yourself and think about why you feel this way.	2.10	0.91	0.48	-0.55
R5	Write down what you are thinking and analyze it.	1.64	0.84	1.20	0.65
R6	Think about a recent situation, wishing it had gone better.	2.32	0.93	0.28	-0.77
R7	Think “Why do I have problems other people don’t have?”	1.96	0.91	0.67	-0.37
R8	Think “Why can’t I handle things better?”	2.19	0.90	0.44	-0.54
R9	Analyze your personality to try to understand why you are depressed.	1.96	0.95	0.70	-0.47
R10	Go someplace alone to think about your feelings.	2.10	0.97	0.57	-0.63
A1	Feeling nervous, anxious or on edge.	1.51	0.75	1.61	2.37
A2	Not being able to stop or control worrying.	1.47	0.75	1.63	2.15
A3	Worrying too much about different things.	1.54	0.82	1.44	1.26
A4	Trouble relaxing.	1.51	0.80	1.57	1.75
A5	Being so restless that it is hard to sit still.	1.43	0.75	1.84	2.83
A6	Becoming easily anno.	1.53	0.81	1.54	1.68
A7	Feeling afraid as if something awful might happen.	1.46	0.78	1.76	2.48
SPD1	Your friends and family complained about your use of the mobile phone.	2.04	1.12	1.07	0.49
SPD2	You have been told that you spend too much time on your mobile phone.	2.11	1.11	0.98	0.33
SPD3	You have tried to hide from others how much time you spend on your mobile phone.	1.68	1.01	1.57	1.91
SPD4	You have received mobile phone bills you could not afford to pay.	1.61	1.00	1.71	2.28
SPD5	You find yourself engaged on the mobile phone for longer period of time than intended.	2.18	1.16	0.86	-0.01
SPD6	You have attempted to spend less time on your mobile phone but are unable to.	2.03	1.10	0.97	0.28
SPD7	You can never spend enough time on your mobile phone.	1.82	1.03	1.22	0.86
SPD8	Feeling anxious and lost when out of range for some time, you become preoccupied with the thought of missing a call.	1.84	1.11	1.32	0.98
SPD9	You find it difficult to switch off your mobile phone.	2.04	1.22	1.08	0.21
SPD10	You feel anxious if you have not checked for messages or switched on your mobile phone for some time.	1.83	1.08	1.29	0.96
SPD11	You feel lost without your mobile phone.	1.87	1.08	1.23	0.83
SPD12	If you don’t have a mobile phone, your friends would find it hard to get in touch with you.	2.31	1.24	0.73	-0.40
SPD13	You have used your mobile phone to talk to others when you were feeling isolated.	2.10	1.20	0.95	0.01
SPD14	You have used your mobile phone to talk to others when you were feeling lonely.	2.20	1.22	0.83	-0.24
SPD15	You have used your mobile phone to make yourself feel better when you were feeling down.	2.34	1.22	0.69	-0.40
SPD16	You find yourself occupied on your mobile phone when you should be doing other things, and it causes a problem.	2.08	1.14	0.92	0.07
SPD17	Your productivity has decreased as a direct result of the time you spend on the mobile phone.	2.05	1.14	0.98	0.19

Notes. $N = 1610$.

Results of the accuracy assessment of the network structure

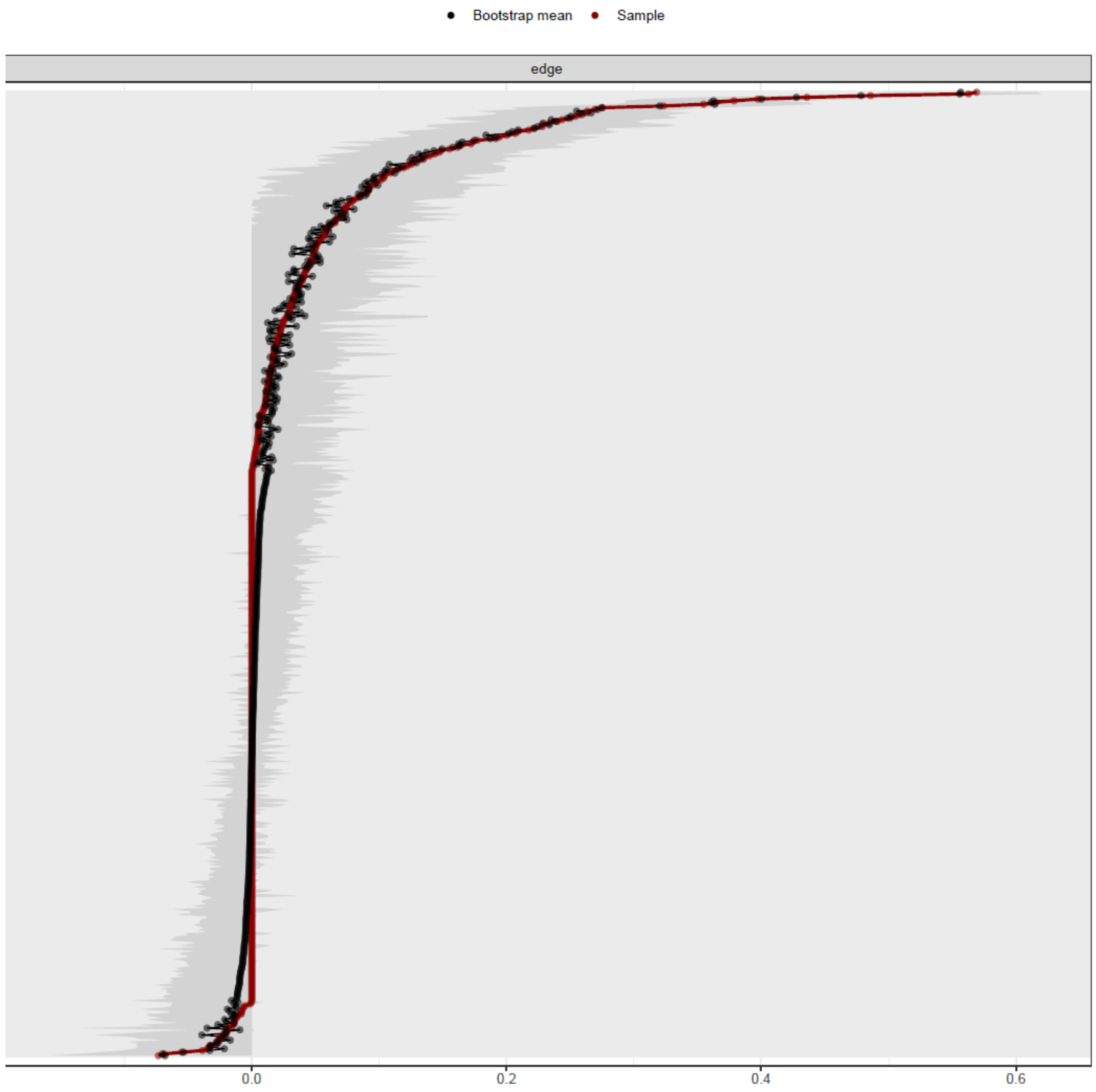

Figure S1. Accuracy of edge weights.

Note: The red line depicts the sample edge weights and the gray bar depicts the bootstrapped confidence interval.

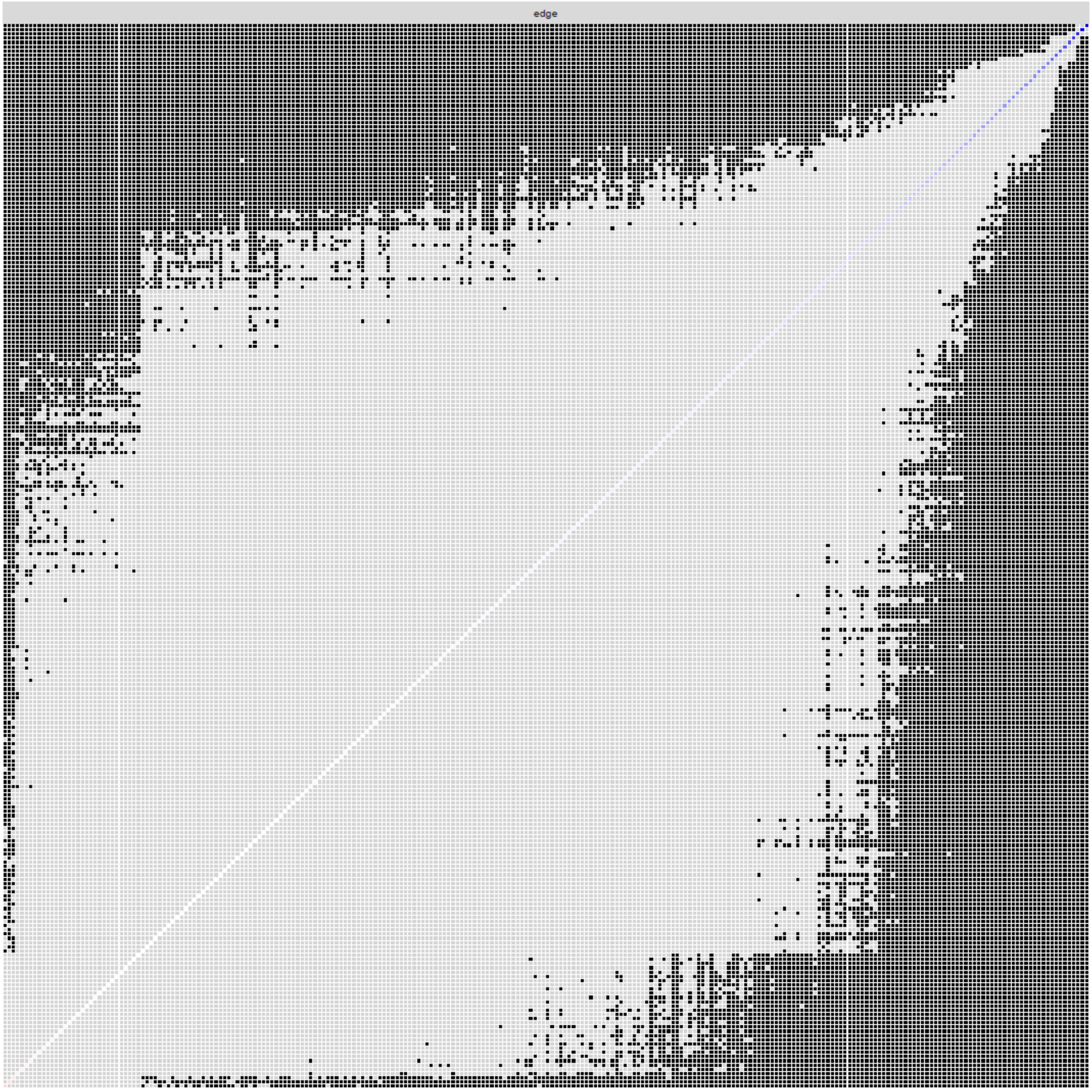

Figure S2. Bootstrapped difference test for edge weights.

Note: Gray boxes indicate edge weights that do not differ significantly from one another, while black boxes indicate edge weights that do differ significantly. Blue and red boxes on the diagonal correspond to edge weights with positive and negative correlations, respectively.

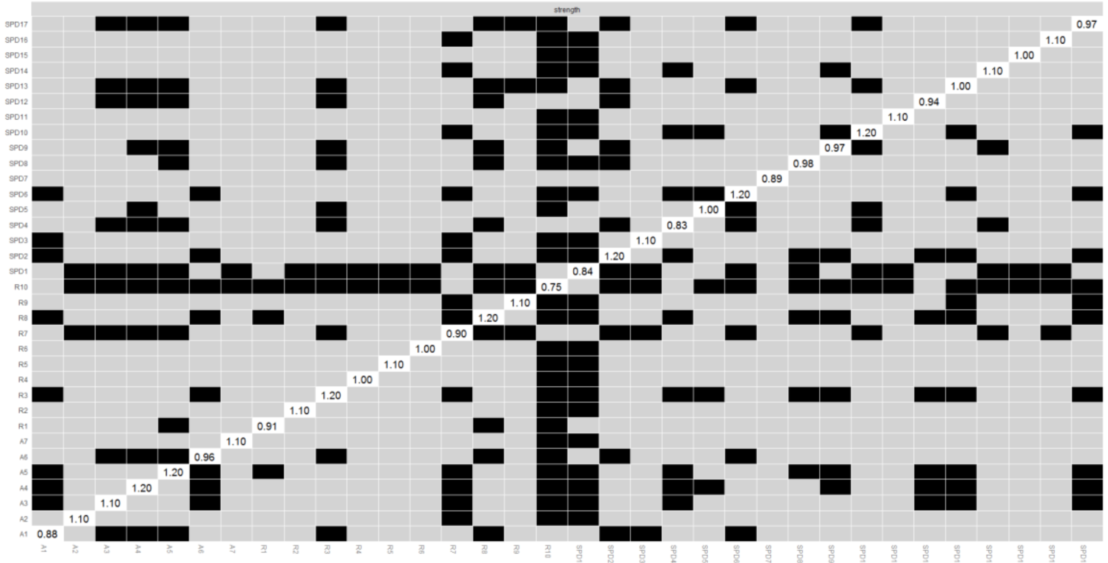

Figure S3. Bootstrapped difference test for node strength.

Note: Gray boxes indicate node expected influences that do not differ significantly from one another, while black boxes indicate node expected influences that do differ significantly.

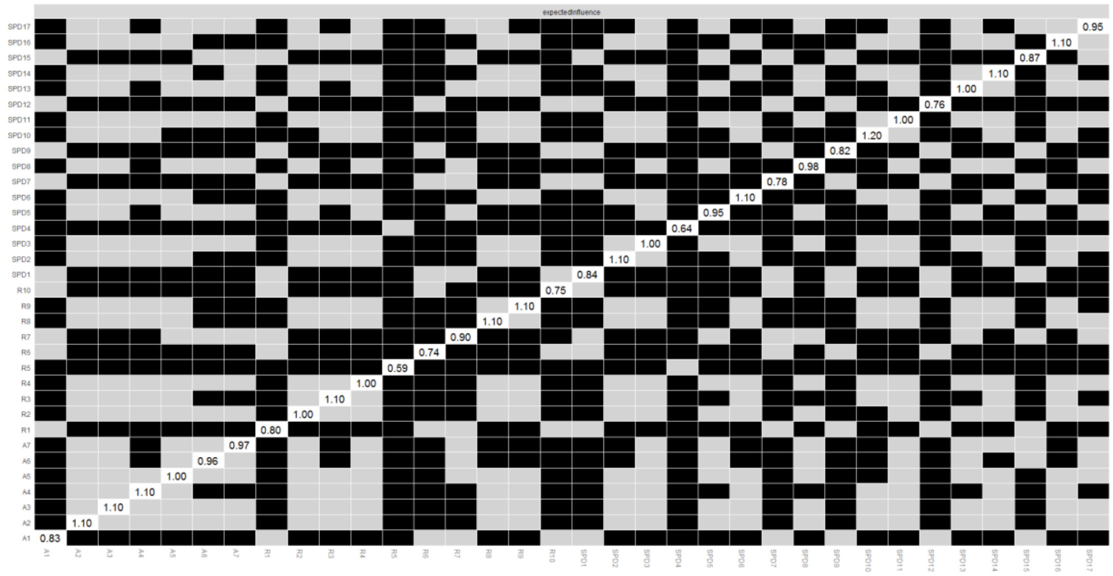

Figure S4. Bootstrapped difference test for node expected influences.
Note: Gray boxes indicate node expected influences that do not differ significantly from one another, while black boxes indicate node expected influences that do differ significantly.

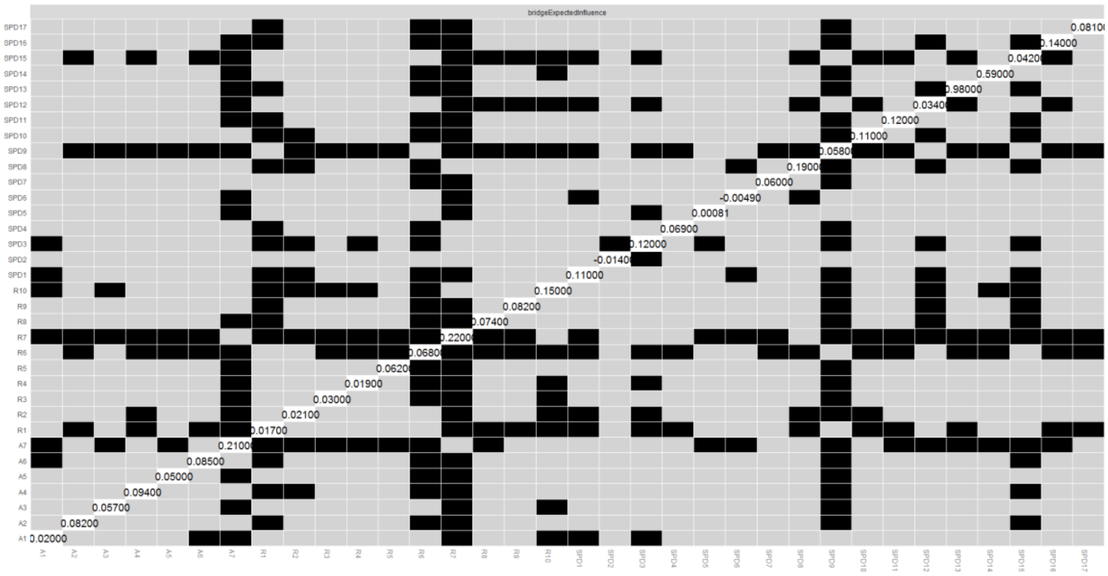

Figure S5. Bootstrapped difference test for node bridge expected influences.

Note: Gray boxes indicate node bridge expected influences that do not differ significantly from one another, while black boxes indicate node bridge expected influences that do differ significantly.
